# Establishing a Cut-Off Value for Zinc Alpha-2 Glycoprotein in Serum as a Potential Biomarker in Children and Adolescents with Obesity

**DOI:** 10.3390/ijms27093773

**Published:** 2026-04-23

**Authors:** Barbara Siewert, Katarzyna Zorena, Anna Sośnicka, Marta Jaskulak, Iwona Beń-Skowronek

**Affiliations:** 1 Environment and Health Scientific Circle, Department of Immunobiology and Environment Microbiology, Medical University of Gdansk, 80-210 Gdansk, Poland; barbara.siewert@gumed.edu.pl; 2 Department of Immunobiology and Environment Microbiology, Faculty of Health Sciences, Medical University of Gdansk, 80-210 Gdansk, Poland; anna.sosnicka@gumed.edu.pl (A.S.); marta.jaskulak@gumed.edu.pl (M.J.); 3 Department of Pediatric Endocrinology and Diabetology, University Children’s Hospital, Medical University of Lublin, 20-093 Lublin, Poland; iwona.ben-skowronek@umlub.pl

**Keywords:** children and adolescents, obesity, metabolic parameters, cut-off value, Least Absolute Shrinkage and Selection Operator (LASSO) model, zinc-α2-glycoprotein (ZAG)

## Abstract

Zinc-α2-glycoprotein (ZAG) is a novel adipokine with a plethora of functions meaningful for the regulation of adipose tissue and insulin sensitivity. Despite research, the role of ZAG in the course of childhood obesity is not fully understood. The aim of this study is to investigate whether the levels of ZAG can be used as a predictive or monitoring biomarker of adolescent obesity. Secondly, to determine the cut-off value of ZAG in blood serum in adolescents with obesity. The study included a group of 77 adolescent patients, including 59 obese patients, and 18 without obesity as healthy control subjects. All study participants had their biochemical parameters assessed by a certified medical laboratory. The recommendations of the Polish Society of Hypertensions were used to assess the blood pressure measurements in each group. ELISA enzyme immunoassays (R&D Systems, Minneapolis, MN, USA) were used to detect serum levels of ZAG. Our study showed that obese children and adolescents have significantly higher body mass, cholesterol, LDL-cholesterol, triglycerides (TG), systolic blood pressure (SBP) and diastolic blood pressure (DBP), but lower serum ZAG levels compared to the healthy control subjects. Furthermore, in our study, we found that median ZAG values were comparable between females and males within the same obesity category (median female ZAG level: 2.84, median male ZAG level: 2.89) and healthy control participants (median female ZAG level: 5.20, median male ZAG level: 4.99). Serum ZAG concentrations were significantly lower in obese participants (2.86 ± 0.40 mg/L) than in the control group (5.10 ± 0.74 mg/L; *p* < 0.001). The multivariable Firth’s logistic regression model, incorporating the selected factors, revealed a significant association between obesity and ZAG. ROC curve analysis indicated strong discriminatory ability of ZAG for identifying obesity, with a proposed cut-off value of 3.62 mg/L. Circulating ZAG level is significantly reduced in children and adolescents with obesity. An important finding of our study is the detection of a cutoff value for serum ZAG levels. Furthermore, the use of the Least Absolute Shrinkage and Selection Operator (LASSO) model can be considered a valuable contribution to defining ZAG as an independent factor associated with obesity.

## 1. Introduction

In recent decades, a global epidemic of obesity in both adults and children has become one of the most significant public health issues. The World Health Organization (WHO) officially recognized obesity as “a chronic condition that requires treatment, promotes the development of other diseases and is associated with increased mortality”. Since the 1980s, in some countries, the combined prevalence has tripled, and the number is prognosticated to rise in the foreseeable future [1]. At present, approximately 155 million school-age children are overweight or obese: 30–45 million are children and adolescents aged 5 to 17, and 22 million are children under 5 years of age [2]. Moreover, afflicted children and teenagers are likely to remain obese in their adulthood [3]. Obese individuals experience immediate, intermediate and long-term consequences that affect their health and mental wellbeing. Patients with childhood or adolescent onset obesity are at risk for dyslipidemia, hypertension, diabetes mellitus, cardiovascular problems, non-alcoholic fatty liver disease, psychosocial disturbances and an impaired quality of life [4,5,6,7,8].

Previous research has shown that both genetic and environmental factors influence the development of obesity in childhood, which can translate into both obesity and complications in children and adolescents [9,10,11,12,13].

Adipose tissue is an endocrinologically active organ; hence, its excess can lead to a dysregulated hormone profile and disrupted interplay between adipokines. Chronic and systemic inflammation are inherent to obesity and, thus, an increase in inflammatory mediators and cytokines, infiltration of the inflammatory cells into adipose tissue further induces pro-inflammatory conditions [14,15].

Of several hundred adipokines, the role of leptin and adiponectin in the progression of obesity has been partially elucidated [16,17,18,19,20,21]. For example, adiponectin is an anti-inflammatory adipokine that sensitizes tissues to insulin [16]. Low adiponectin levels are linked to increased adiposity, impaired glucose tolerance, systemic inflammation, and early metabolic dysfunction, suggesting that diminished adiponectin contributes directly to the development of insulin resistance risk [17,18,19]. Leptin, on the other hand, is an adipocyte-derived hormone primarily involved in appetite regulation and energy expenditure via inhibiting the synthesis and release of neuropeptide Y (NPY) in the arcuate nucleus (ARC) [20]. Recent studies have shown that leptin levels are elevated in obese children due to increased fat mass and the development of leptin resistance, leading to reduced satiety and impaired metabolic regulation [21]. However, the role of ZAG has not been fully explained and research results are inconsistent [22,23,24]. Thus, Martínez-Navarro et al., examining serum ZAG levels in obese children, found no statistically significant differences between obese and non-obese children [22]. In other studies, overweight and obese children had significantly lower ZAG levels than their normal-weight peers [23]. Our latest studies in adults [24] showed that the mean concentration of ZAG in the group with normal body weight was 791.46 ng/mL; in the overweight group, it was 680.2 ng/mL (14.06% lower than in patients with normal weight); and in the group with obesity, the mean concentration was 380.65 ng/mL (56.16% lower than in the group with normal body weight) [24].

Zinc-α2-glycoprotein is a novel adipokine that has been implicated in the regulation of adipose tissue, insulin sensitivity and lipid mobilization [25,26]. Recent research suggests that ZAG may also exhibit anti-inflammatory properties and its adaptive expression patterns may therefore act as a protective agent against obesity and related comorbidities [27,28].

Therefore, in the present study, we assessed serum ZAG concentrations in children and adolescents with obesity and compared them with those of healthy controls.

The aim of this study was to determine whether ZAG levels can be used as a predictive or monitoring biomarker of obesity in adolescents. Furthermore, the study aimed to determine the cut-off value for serum ZAG concentration in children and adolescents.

## 2. Results

### 2.1. Clinical Characteristics of Patients with Obesity and Healthy Control Subjects

The study population consisted of two groups: adolescents with obesity (n = 59) and healthy controls with normal body weight (n = 18). The criterion for obesity classification was a body mass index (BMI) value above the 97th percentile for each age and sex based on the percentile charts developed by the Institute of Mother and Child/WHO.

Table 1 summarizes the characteristics of the study population, stratified by obesity status. Our study group included 77 participants, of whom 59 were classified as obese patients and 18 as healthy control subjects. Age did not differ significantly between groups (*p* = 0.073). BMI values differed significantly between groups 36 ± 5 vs. 18 ± 2; *p* < 0.001. In both the obese and healthy groups, pubertal development was assessed using the Tanner scale. The distribution of maturity phases in relation to the groups differed statistically significantly (*p* = 0.035).

Differences in metabolic health were also pronounced in our cohort. Fasting glucose levels were lower among participants with obesity (81 ± 8 mg/dL) compared to the non-obese group (93 ± 5 mg/dL; *p* < 0.001). OGTT results were performed in the obese group only due to protocol and policy constraints. However, the median value of the oral glucose tolerance test (OGTT) at 120 min was equal to 112, while the homeostatic model assessment (HOMA) reached the median of 3.0 in both obese children and healthy control subjects in our study groups. The study also demonstrated that the level of ZAG in blood serum was lower in adolescents with obesity, reaching 2.86 ± 0.40 mg/L, *p* < 0.001, compared to 5.10 ± 0.74 mg/L in the group of healthy control subjects. The differences were statistically significant (*p* < 0.001) (Table 1).

**Figure 1:**
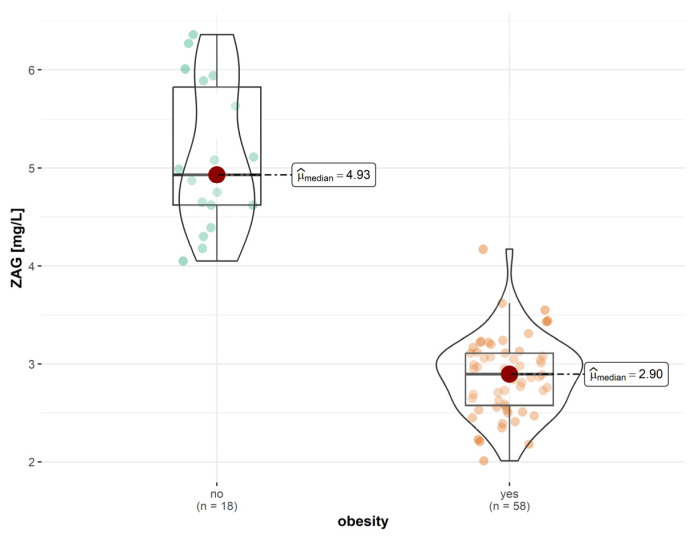
Visual representation of serum ZAG levels in obese and healthy controls. Mann–Whitney U test was used to examine differences in ZAG levels between obese (median = 2.90) and healthy control subjects (median: 4.93), W = 1043.00, *p* = 2.02 × 10^−10^. Abbreviation: ZAG—zinc alpha-2 glycoprotein.

The Mann–Whitney U test showed that healthy control subjects with a median score of 4.93 exhibited significantly higher ZAG levels compared to those with obesity, with a median = 2.90 (W = 1043.00, *p* = 2.02 × 10^−10^) (Figure 1). The rank-biserial correlation indicated a very large effect size (rank biserial = 1.00, 95% CI [1.00, 1.00]), suggesting a strong and consistent association between obesity status and ZAG levels. Statistical analysis showed that the serum ZAG levels are significantly lower in patients with obesity compared to the healthy control group (Figure 1).

### 2.2. Serum Zinc Alpha-2 Glycoprotein Levels in Obese Patients Depending on Sex

**Figure 2:**
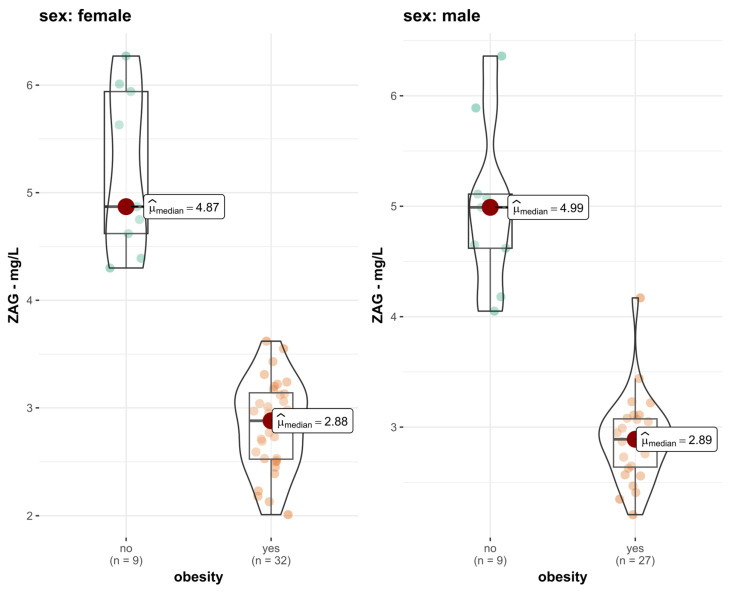
Visual representation of ZAG levels in obese patients and the healthy control group, divided by sex. Mann–Whitney U test was used to examine differences in ZAG levels between obese (median female ZAG level: 2.84, median male ZAG level: 2.89) and healthy control participants (median female ZAG level: 5.20, median male ZAG level: 4.99.

Figure 2 presents how serum ZAG concentrations differed according to obesity status among male and female groups. The distribution plots show a downward trend in ZAG levels in the female and male obese groups, with very limited overlap between the male obese and male non-obese groups. Among females, median ZAG levels were markedly higher in healthy control participants (median = 4.87 mg/L; n = 9) compared to obese subjects (median = 2.88 mg/L; n = 32). A similar trend holds true for the male group, where healthy control boys exhibited higher median ZAG levels (median = 4.99 mg/L; n = 9) than their obese counterparts (median = 2.89 mg/L; n = 27). These findings suggest that obesity status, rather than sex, is the primary determinant of circulating ZAG levels in study groups.

### 2.3. Correlations Between Serum Levels of ZAG and Other Clinical Parameters in the Obesity Patients

**Figure 3:**
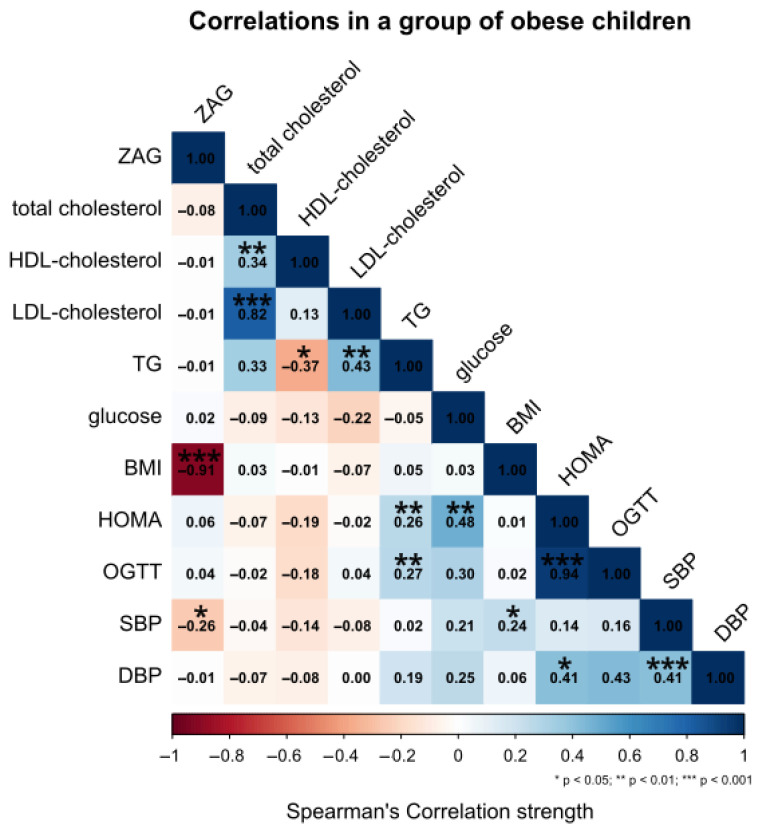
The relationship between serum levels of ZAG and other clinical parameters in the obese group. The level of statistical significance is marked as asterisk.

Spearman’s correlation analysis in the obese group revealed a very strong negative correlation between ZAG and BMI (r = −0.91, p < 0.001). Moreover, a statistically significant negative correlation was detected between ZAG and systolic blood pressure (SBP) (r = −0.26, p < 0.05), while no significant associations were observed between ZAG and other parameters such as lipid fractions, glucose, HOMA-IR or OGTT, TG, and diastolic blood pressure (DBP) (Figure 3).

### 2.4. Multivariate Logistic Regression with LASSO (Least Absolute Shrinkage and Selection Operator) Penalty, and a Firth’s Penalized Likelihood Logistic Regression Model

A logistic regression model was used to identify independent risk factors for obesity among variables including ZAG, age, total cholesterol, LDL-cholesterol, HDL-cholesterol, TG, BP, glucose, HOMA-IR, and pubertal phase according to Tanner scale. Due to the low event-per-variable ratio (EPV ≈ 1.4), a Stability Selection procedure based on a non-parametric bootstrap (2000 iterations) was employed to mitigate post-selection inference bias and ensure the robustness of the findings.

In each bootstrap iteration, automated variable selection was performed using LASSO (Least Absolute Shrinkage and Selection Operator) penalized logistic regression. The optimal penalty parameter (λ) was determined via 5-fold cross-validation using the minimum deviance criterion (λmin). Variables selected by the LASSO algorithm in at least 50% of all bootstrap repetitions were classified as stable predictors. The stability selection process, based on 2000 bootstrap iterations, identified three variables that exceeded the pre-defined 50% inclusion frequency threshold at the λmin penalty level (Table 2).

To obtain final effect estimates for the selected stable variables, a Firth’s penalized likelihood logistic regression model was constructed. This method was chosen to provide unbiased estimates and reliable 95% confidence intervals (CI) in the presence of a low number of events and to prevent issues related to data separation.

The highest stability was observed for ZAG (99.9%), Glucose (75.65%) and HDL-cholesterol (54.1%). The final multivariable Firth’s logistic regression model, incorporating the selected factors, revealed a significant association between obesity and ZAG (Table 3).

The analysis conducted using Firth’s penalized logistic regression demonstrated that the developed model is significantly superior to the null model, as confirmed by both the likelihood ratio test (LR = 57.41; *p* < 0.0001) and the Wald test (*p* = 0.0098). The ZAG variable emerged as the key predictor, exhibiting a strong, statistically significant association with the odds of the event occurring (*p* < 0.0001).

A very high intercept (Intercept = 24.27) and an odds ratio for the ZAG variable approaching zero (OR ≈ 0) indicate the presence of near-complete separation (also known as monotone likelihood). Specifically, the ZAG variable differentiates obese patients from healthy controls almost perfectly, where each unit increase is associated with a radical, over 99% reduction in the odds of the studied endpoint.

The remaining variables included in the model, following LASSO selection—namely HDL and glucose levels—did not reach the threshold of statistical significance (*p* > 0.05), suggesting that their contribution to the multivariable model variance is marginal. Their inclusion in the final specification, despite the lack of significance, may stem from the optimization of the penalty criterion within the LASSO procedure; however, in a clinical context, their impact remains unconfirmed and likely represents statistical noise within this particular study sample.

### 2.5. Determination of the Cut-Off Value for ZAG in Blood Serum in the Study Group

**Figure 4:**
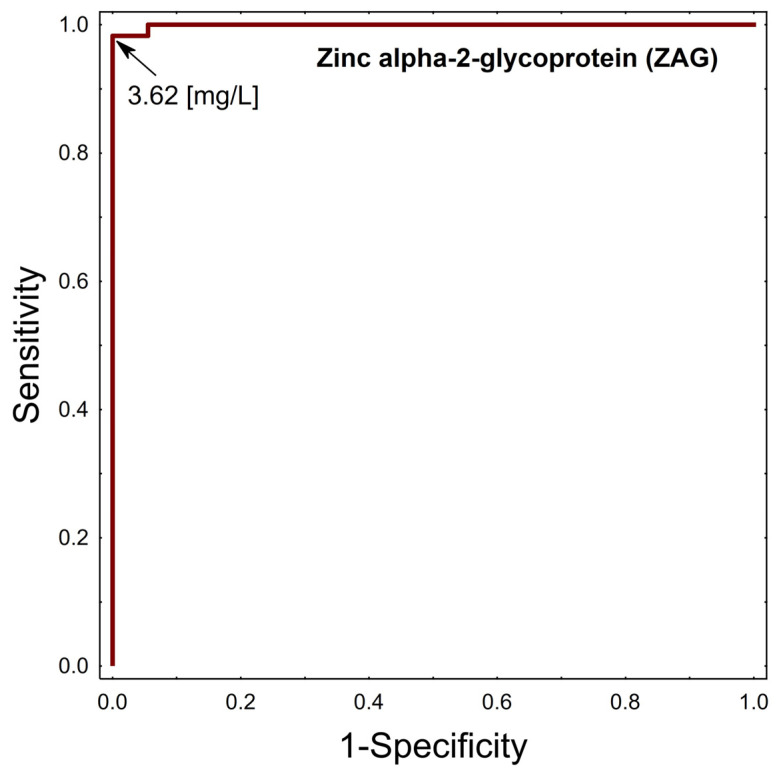
AUC_ROC_ for serum ZAG.

The threshold serum ZAG concentrations that had discriminatory ability to predict the occurrence of obesity were calculated using receiver operating characteristic (ROC) curve analysis and were 3.62 mg/L. The area under the ROC (AUC_ROC_) was 0.999, and its population value was in the range 0.996–1.000 [*p* < 0.0001], shown in Figure 4. The obtained AUC value indicates an almost perfect discriminatory ability of the investigated parameter. The confidence interval is very narrow and includes values close to 1.0, confirming the high precision of the estimate. Statistical testing demonstrated a significant difference compared with the reference value of 0.5 (z = 308.324; *p* < 0.0001).

## 3. Discussion

Our study revealed significantly higher body mass index, LDL-cholesterol, TG, SBP, and DBP in obese children and adolescents compared to the group of healthy control subjects. The fasting glucose levels were lower in obese participants compared to the healthy control group. Our glucose results are consistent with the mechanism of hyperinsulinemia, i.e., children and adolescents with obesity who do not have diabetes often present with lower fasting glucose levels due to hyperinsulinemia [29]. After meals, glucose levels may also be reduced as a result of increased insulin secretion. However, in some children, insulin resistance develops. This process can lead to prediabetes (impaired glucose tolerance) and eventually progress to diabetes [29,30]. In fact, the oral glucose tolerance test [OGTT] is the gold standard for the diagnosis of this high-risk condition for the development of T2D and cardiovascular disease. Young people with obesity and 2hPG values in the range of 120–139 mg/dL already have impaired β-cell function relative to insulin sensitivity, which increases the risk of developing Impaired Glucose Tolerance (IGT) and type 2 diabetes in the future [31]. It should be borne in mind that the OGTT test has drawbacks. The most critical disadvantages of OGTT are its poor reproducibility and the lack of validation in children. Other relevant issues are the need for an overnight fast and the length of the test, which could be demanding, particularly for younger children, and associated nausea can occur in a subset of individuals [32]. Our study also demonstrated that the level of ZAG in blood serum was lower in adolescents with obesity, reaching 2.86 ± 0.40 mg/L, *p* < 0.001, compared to 5.10 ± 0.74 mg/L in the group of healthy control subjects. Although our results are consistent with the known physiological interplay between adipokines and metabolic regulation in adults [24,26,33], they offer an interesting insight into the same relationships but in the adolescent, often overlooked, population. The strong negative relationship between ZAG and BMI suggests that ZAG secretion is significantly reduced in states of increased adiposity. This finding suggests that ZAG may play a compensatory or protective role against excessive adiposity or is involved in compensatory mechanisms that support metabolic homeostasis: it induces lipolysis and inhibits lipogenesis. Not only can the analysis of obesity and overweight-related ZAG level changes give an insight into its role in the metabolic abnormalities, but also the assessment of ZAG contribution in the opposite disorders, such as anorexia nervosa and cachexia [34]. In a study concerning teenage anorexia nervosa subjects, ZAG was found to rise with the deterioration of nutritional status. The authors found that patients with anorexia nervosa had significantly higher mean ZAG concentration than obese patients [34].

In a obese adults study conducted by Selva et al., ZAG levels were found to be inversely associated with body weight and to stimulate lipolysis in individuals with obesity [33]. Our previous study conducted on adults showed a 56.16% lower mean ZAG concentration in the obese group compared to people with normal body weight [24]. Moreover, Liu et al. demonstrated that serum ZAG levels were significantly lower in patients with premature coronary artery disease [26].

Several experimental studies partially explain the underlying action of ZAG. Elattar et al. suggest that ZAG promotes the browning of white adipose tissue, thus increasing lipolysis [35]. This process is driven by activation of protein kinase A (PKA) and p38 mitogen-activated protein kinase (MAPK) pathways, and through these mechanisms, ZAG upregulates a range of genes involved in lipolysis, including UCP-1 [36].

Moreover, increased ZAG expression was found to promote lipid breakdown while suppressing lipid synthesis. This is reflected by reduced mRNA levels of fatty acid synthase (FAS), acetyl-CoA carboxylase, and diacylglycerol acyltransferase, alongside elevated expression of hormone-sensitive lipase [37].

Interestingly, in our study, we found that median ZAG values were comparable between females and males within the same obesity category (median female ZAG level: 2.84, median male ZAG level: 2.89) and healthy control participants (median female ZAG level: 5.20, median male ZAG level: 4.99), implying that obesity status, rather than sex, is the primary determinant of serum ZAG levels in our study population.

A logistic regression model was used to identify independent risk factors for obesity among variables including ZAG, age, total cholesterol, LDL-cholesterol, HDL-cholesterol, TG, BP, glucose, HOMA-IR, and pubertal phase according to Tanner scale. The stability selection process, based on 2000 bootstrap iterations, identified three variables that exceeded the pre-defined 50% inclusion frequency threshold at the λmin penalty level. The highest stability was observed for ZAG (99.9%), Glucose (75.65%) and HDL-cholesterol (54.1%). The final multivariable Firth’s logistic regression model, incorporating the selected factors, revealed a significant association between obesity and ZAG. Finally, to determine the cutoff value for ZAG, we used Receiver Operating Characteristic (ROC) curve analysis. ROC curve analysis helps visualize and select optimal threshold values that will ensure the highest decision accuracy. We believe that the key finding of our study is the detection of a cutoff value of 3.62 mg/L for serum zinc-alpha-2-glycoprotein in children with obesity. To our knowledge, this study is one of the first to propose a potential diagnostic cutoff value for serum ZAG levels in children with obesity. The ROC curve analysis demonstrated a very high discriminatory performance of ZAG (AUC = 0.999), with a proposed cut-off value of 3.62 mg/L. However, such a near-perfect classification should be interpreted with caution, as it may reflect overfitting due to the relatively small sample size and group imbalance. Importantly, the cut-off value was derived and evaluated within the same dataset and therefore requires validation in independent cohorts before any clinical application can be considered.

Although the action of ZAG has not been fully elucidated, there is strong evidence that supports its role in many biochemical and physiological regulations. ZAG is reported to regulate the glucose levels in multiple mechanisms: increasing the urinary glucose excretion, decreasing the maximal plasma glucose and insulin levels in oral glucose tolerance tests and promoting the transfer of glucose into skeletal muscle and adipocytes. The underlying mechanism is thought to be the binding of ZAG to b2 and b3 but not b1 adrenergic receptors and their activation in white and brown adipose tissues, which leads to an increase in cAMP [38].

Unlike leptin, ZAG levels do not provide relevant explanations to disrupted glucose homeostasis and insulin resistance, suggesting that ZAG’s primary metabolic relevance lies in body fat regulation rather than direct modulation of glucose–insulin homeostasis in the adolescent population. Nevertheless, the standardization of assays, establishment of reference ranges, and validation in large, prospective cohort studies are needed for reaching the official recognition of the relevance of ZAG as a validated clinical parameter. Further research to elucidate the pathways through which ZAG interacts with other adipokines and influences metabolic homeostasis in pediatric, adolescent and adult populations is encouraged.

### Limitations

The authors of this article are aware of certain limitations of the presented study results. First, the study group should have been larger. Furthermore, there is a lack of in-depth interview data regarding genetic predisposition, i.e., whether parents and/or grandparents were obese. There is no data regarding the physical activity or nutritional status of children and adolescents with obesity. In the future, the data should be expanded to include a larger study sample and detailed interview data regarding nutritional status and physical activity.

## 4. Materials and Methods

The study included a group of 77 adolescent patients, including 59 patients with obesity, and the control group consisted of 18 healthy control subjects. The study and control groups were created based on cooperation with the Department of Pediatric Endocrinology and Diabetology, with the Endocrinology and Metabolic Laboratory at the University Children’s Hospital in Lublin. The study included anthropometric measurements such as height measurement using a stadiometer and weight measurement using a medical scale.

Each participant had their blood pressure assessed according to the recommendations of the Polish Society of Hypertension. Blood pressure was measured in resting conditions, after the child had rested for at least 5 min in a sitting position. Values above 95 pc were defined as elevated BP based on the guidelines for screening and management of high blood pressure in children and adolescents [39].

Serum total cholesterol, low-density lipoprotein (LDL) and high-density lipoprotein (HDL) cholesterol levels were assayed by the ARCHITECT cSystem and AEROSET, Abbott, Wiesbaden, Germany. The criterion for qualifying for the study group was body mass index (BMI) calculated on the basis of anthropometric measurements, which was then applied to the OLA and OLAF centile charts [40]. The exclusion criterion for the study was the lack of consent from patients or parents of patients, the occurrence of congenital genetic diseases causing metabolic syndrome from early childhood, or taking medications affecting glucose-lipid metabolism.

The study received a positive opinion from the Bioethics Committee No. (KE-0254/286) at the Medical University of Lublin.

### 4.1. Laboratory Analyses

For each patient, anthropometric measurements were taken using the same scales. Height measurements were performed with an accuracy of 0.5 cm and body weight measurements with an accuracy of 0.1 kg. The patients wore minimal clothing, were barefoot, and were commanded to stand still. The body mass index (BMI) was calculated according to the following formula: BMI = body weight (kg)/height (m^2^).

### 4.2. Testing of Basic Parameters in Blood Serum

The level of glucose exponents and lipid profile (total cholesterol, triglycerides, high-density lipoprotein, low-density lipoprotein) were determined in the Medical Analysis Laboratory at the University Children’s Hospital in Lublin. Roche technology was used to determine the above parameters according to standard procedures: glucose was determined using the hexokinase method (ELECYC-Cobas c311 Roche Diagnostics GmbH, Mannheim, Germany), while the lipid profile was determined using the enzymatic-colorimetric method (ELECYC-CobasIntegra 400plus, Roche Diagnostics GmbH, Mannheim, Germany, and HDL—ELECYC-Cobasc311, Roche Diagnostics GmbH, Mannheim, Germany).

Blood samples were collected from 77 children, and were immediately placed on ice, clarified by centrifugation at 3000× *g* for 15 min at 4 °C, and kept frozen at −80 °C until assayed. Serum levels of ZAG were measured by ELISA enzyme immunoassays (R&D Systems, Minneapolis, MN, USA) according to the manufacturer’s protocol. In total, 100 µL of serum was used for each assay. The level of absorbance was measured on an automatic plate reader (ChroMate 4300, Awareness Technology, Inc., Palm City, FL, USA). The manufacturer’s guidelines were followed when creating standard curves.

### 4.3. Statistical Analyses

The statistical analysis was performed using R software, version 4.3.3. The significance level was set at α = 0.05, and all tests were two-tailed. Numerical data were expressed as the mean and standard deviation (SD) for variables with a normal distribution, or as the median and interquartile range (IQR) for variables with a non-normal distribution.

The Student’s *t*-test was used to compare two independent groups, or the Mann–Whitney U test when the assumption of normality was not met. Relationships between two continuous variables were assessed using Pearson’s correlation coefficient for normally distributed variables, or Spearman’s correlation coefficient; otherwise, logistic regression was used to identify independent risk factors for obesity among the examined variables.

Due to the low event-per-variable ratio (EPV ≈ 1.4), a Stability Selection procedure based on a non-parametric bootstrap (2000 iterations) was employed to mitigate post-selection inference bias and ensure the robustness of the findings.

In each bootstrap iteration, automated variable selection was performed using LASSO (Least Absolute Shrinkage and Selection Operator) penalized logistic regression. The optimal penalty parameter (λ) was determined via 5-fold cross-validation using the minimum deviance criterion (λmin). Variables selected by the LASSO algorithm in at least 50% of all bootstrap repetitions were classified as stable predictors.

To obtain final effect estimates for the selected stable variables, a Firth’s penalized likelihood logistic regression model was constructed. This method was chosen to provide unbiased estimates and reliable 95% confidence intervals (CI) in the presence of a low number of events and to prevent issues related to data separation. All analyses were performed in R (version 4.3.3) using glmnet (LASSO), logistf (Firth regression), and boot (bootstrap) packages.

To determine the discriminating threshold value, ROC curve analysis and calculation were performed usingSTATISTICA version 13.3. Receiver operating characteristic (ROC) curve analysis was performed to evaluate the ability of serum ZAG concentrations to discriminate between obese and non-obese participants. The optimal cut-off value was determined using the Youden index. The area under the ROC curve (AUC) was calculated as a measure of diagnostic performance.

## 5. Conclusions

Our study demonstrated lower levels of zinc alpha-2 glycoprotein and established a cutoff value for this serum concentration in children with obesity. Furthermore, the use of the LASSO model can be considered a valuable contribution to defining ZAG as an independent factor associated with obesity. Further studies with larger numbers of children and adolescents with obesity are warranted.

## Figures and Tables

**Table 1:** Clinical characteristics of patients with obesity and healthy control subjects.

Parameters	Obese Patients N = 59	Healthy Control Subjects N = 18	*p*-Value
Age (years)	15.00 (13.50, 16.00)	13.50 (8.00, 15.00)	0.073 ^1^
Sex (female/male)	32/27	9/9	Ns
Pubertal phase according to the 5-point Tanner scale (n)			0.035
1	2	3	
2	4	1	
3	11	3	
4	12	5	
5	31	2	
BMI	36 ± 5	18 ± 2	<0.001
Glucose [mg/dL]	81 ± 8	93 ± 5	<0.001
HOMA-IR	3.00 (3.00, 5.00)	3.00 (2.00, 3.00)	0.003 ^1^
OGTT [mg/dL]	112 (100, 126)	NA (NA, NA)	-
Total cholesterol (mg/dL)	157 (139, 176)	157 (142, 174)	0.9 ^1^
LDL-cholesterol (mg/dL)	110 ± 30	87 ± 19	0.006
HDL-cholesterol (mg/dL)	39 (33, 46)	54 (43,62)	<0.001 ^1^
TG [mg/dL]	98 (79, 132)	77 (65, 99)	0.038 ^1^
SBP [mmHg]	128 ± 16	110 ± 13	<0.001
DBP [mmHg]	77 ± 10	64 ± 9	<0.001
ZAG [mg/L]	2.86 ± 0.40	5.10 ± 0.74	<0.001

^1^ Median (Q1, Q3); Mean (±SD); Continuous data are presented as mean ± standard deviation. Abbreviation: BMI—body mass index [kg/m^2^], HOMA—homeostatic model assessment, LDL—low-density lipoproteins, HDL—high-density lipoprotein, TG—triglycerides, SBP—systolic blood pressure, DBP—diastolic blood pressure, OGTT—oral glucose tolerance test at 120 min, ZAG—zinc alpha-2 glycoprotein, NA—not tested.

**Table 2:** Results of the stability selection procedure for independent variables in the obesity model (Bootstrap LASSO, 2000 iterations).

Predictor/Variable	Selection Count (n)	Inclusion Probability (%)
ZAG	1.998	99.9
Glucose	1.513	75.65
HDL-cholesterol	1.082	54.1
HOMA-IR	947	47.35
LDL-cholesterol	740	37
Age	714	35.7
Pubertal phase according to Tanner scale	508	25.4
Blood pressure	262	13.1
cholesterol	120	6
TG	91	4.55

Abbreviation: ZAG—zinc alpha-2 glycoprotein, HDL—high-density lipoprotein, LDL—low-density lipoproteins, TG—triglycerides, SBP—systolic blood pressure, DBP—diastolic blood pressure, HOMA—homeostatic model assessment.

**Table 3:** Parameters of the final Firth’s penalized logistic regression model for obesity risk factors.

Variable	Odds Ratio (OR, 95% CI) ^1^	Chi-Square (χ^2^)	*p*-Value
(Intercept)	34,750,868,115.2565 (5641.0102–4.39102949284515 × 10^30^)	12.692	0.0004
ZAG [mg/L]	0.0045 (0–0.1527)	29.918	0.0000
HDL-cholesterol [mg/dL]	1.0516 (0.9589–1.2667)	1.064	0.3024
Glucose [mg/dL]	0.9381 (0.6476–1.1806)	0.314	0.5752

Abbreviation: ZAG—zinc alpha-2 glycoprotein, HDL—high-density lipoprotein, ^1^ Profile Likelihood Confidence Intervals. Likelihood ratio test = 57.41 on 3 df, *p* < 0.0001, n = 71. Wald test = 11.38 on 3 df, *p* = 0.0098.

## Data Availability

The original contributions presented in this study are included in the article. Further inquiries can be directed to the corresponding author.

## Abbreviations

The following abbreviations are used in this manuscript:

ZAG	Zinc-α2-glycoprotein
TG	triglycerides
SBP	systolic blood pressure
DBP	diastolic blood pressure
WHO	World Health Organization
NPY	Neuropeptide Y
ARC	Arcuate nucleus
HDL	High-density lipoprotein
LDL	Low-density lipoprotein
BMI	Body mass index
ROC	Receiver operating characteristic
SD	Standard deviation
IQR	Interquartile range
OGTT	Oral glucose tolerance test
HOMA	Homeostatic Model Assessment
PKA	protein kinase A
MAPK	p38 mitogen-activated protein kinase
FAS	fatty acid synthase
GLUT4	glucose transporter 4
LASSO	Least Absolute Shrinkage and Selection Operator
